# Non-physician Clinicians – A Gain for Physicians’ Working in Sub-Saharan Africa: Comment on "Non-physician Clinicians in Sub-Saharan Africa and the Evolving Role of Physicians"

**DOI:** 10.15171/ijhpm.2016.110

**Published:** 2016-08-15

**Authors:** Delanyo Dovlo, Ibiso Ivy King-Harry, Kevin Ousman

**Affiliations:** ^1^World Health Organization (WHO) Africa Region Office, Brazzaville, Congo.; ^2^SUN Business Network, Global Alliance for Improved Nutrition, Abuja, Nigeria.; ^3^Department of Health System Policies and Operations, World Health Organization Regional Office for Africa (AFRO) Brazzaville, Congo.

**Keywords:** Non-physician Clinicians (NPCs), Physician Training, Nurses, Task-Shifting, Health Workforce, Training, Leadership

## Abstract

The changing demands on the health sectors in low- and middle-income countries especially sub-Saharan African countries continue to challenge efforts to address critical shortages of the health workforce. Addressing these challenges have led to the evolution of "non-physician clinicians" (NPCs), that assume some physician roles and thus mitigate the continuing shortage of doctors in these countries. While it is agreed that changes are needed in physicians’ roles and their training as part of the new continuum of care that includes NPCs, we disagree that such training should be geared solely at ensuring physicians dominated health systems. Discussions on the workforce models to suit low-income countries must avoid an endorsement of a culture of physician focused health systems as the only model for sub-Saharan Africa (SSA). It is also essential that training for NPCs be harmonized with that of physicians to clarify the technical roles of both.

Non-physicians, playing some roles traditionally reserved for physicians, have existed in various forms in sub-Saharan African countries even before independence from colonial rule over 50 years ago.^1,2^ Various health cadres, some new, some already established ones such as nurses, have played such delegated and at times assumed roles, with varying scopes of practice and within varied regulatory contexts^3-7^; whether they were legally permitted or not to carry out these roles. Such cadres have been used because all the low- and middle-income countries in sub-Saharan Africa (SSA) have never had sufficient numbers of the more traditional types of health professions and have therefore, developed various coping mechanisms to fill the gaps. These coping mechanisms are not new and continue to be implemented to this day, though they may now get much needed attention as an important and integral component of the health workforce. Any discussion of non-physician practitioners, clinicians and task-shifting, is, therefore, an important part of the planning of human resources for health (HRH) in poorer countries.

The article by Eyal et al,^8^ does however take an interesting angle to this issue of physician substitution and roles replacement. It suggests that this phenomenon is a relatively recent and perhaps troubling phenomenon for physicians and therefore, requiring that physicians’ training be changed in order to continue to ensure a superior role for physicians over other cadres including the so-called non-physician clinicians (NPCs).

The article defines NPCs generally as being health workers with fewer skills than physicians but who have “more skills than nurses,” and this is an interesting definition, that assumes a common global scope of practice and an absence of varied skill ranges that each cadre may gain through additional and specialized training, gained experience, and other development activities.

Many countries have expanded the development of such cadres, but the numbers of physicians trained, while still relatively low, have not remained stagnant,^9^ with very significant increases in the number of medical schools in SSA**.**^10^ The article presents an acceptable analysis of a growth of physician assistants in SSA including a description of the expansion in the numbers of cases being seen by NPCs in Tanzania, which may well be an issue of improved access to all services or salary motivation.^11^ There is a growing demand for NPCs with the supply not being met even in developed countries,^12,13^ there is, therefore, a need to look into policies, and relax regulations where necessary to support health reforms.^14^

World Health Organization Regional Office for Africa’s (WHO AFRO’s) health workforce observatory data from 2010 shows us that while 20 out of 36 countries deemed with HRH crisis in the African region, had made some progress between 2005 and 2010, 10 countries still face critical shortages with density varying from 0.16 to 0.47 of doctors, nurses and midwives per 1000 population, which is far below the recommended minimum density of 2.3 per 1000 population, not to mention the shortage of other categories of health workers.^15,16^ Consequently, almost no country in SSA will have the ratio of 2.5/10 000 population of core health workers, being the threshold needed to be able to deliver and sustain certain key services, including those for HIV, tuberculosis (TB), and Malaria. These three diseases, among others, have received significant resources through various global health initiatives (GHIs), though sometimes inequitably funded; and over time have required health sectors to delegate key physician tasks to non-physicians in order to attain effective coverage of interventions.^5^

The reasons for decentralizing these roles and tasks are multi fold. Government budget constraints meant NPCs were cheaper to train as the article indicates and they also filled persistent gaps in health workforce needs caused by perennial lack of funding for their employment and their loss through their recruitment by richer countries. There is no doubt that these changes and other contextual factors should shift the thinking of health sector planners on how to utilize physicians (and indeed other cadres) differently and more effectively. However, this should not necessarily become an opportunity to ensure physician hegemony on the delivery of services, but to enrich existing roles so that physicians can focus on playing more sophisticated service delivery roles.

We have struggled to understand the notion of “*NPC-based health systems*” and the panic it seems to generate which necessitated the theme of a need to change physicians’ roles. It, therefore, seems to suggest that a “normal” health system is one that must be dominated by physicians, and therefore, having other cadres with enhanced roles perhaps creates an “undesirable” health system. In many health sectors facing HRH challenges, new cadres’ roles and functions usually aim to give adequate independence of functions, of course within a certain measure of supervision and oversight. As with all health cadres, for NPCs to be effective, much emphasis should also be placed on allocation of resources for their training and retraining as this is shown to improve healthcare.^17^

The article rightly understands that having NPCs in both developing and developed country contexts does not deny the usefulness of physicians roles (physicians roles section) and that these include superior clinical roles and “mentoring” of other cadres. There is also an important and continuing role of supporting and mentoring junior doctors which does not get a mention.^18,19^ Doctors also need supervision and in examples from Timor-Leste, the supervision of doctors and other health workers is part of the responsibility of district-level health managers and health management teams.^20^

Physicians sometimes lead or work in multi-disciplinary health teams and this should not only be expected in “NPC-based health systems” as the article suggests. In many rural health facilities in Africa today, new and junior doctors are possibly being mentored and guided by nurses and other cadres (including NPCs). Being a doctor should not “genetically” give leadership and dominance over every cadre and the authors have correctly noted that “integrated and interdependent health service delivery chains are an imperative everywhere”; however, they then say that “*but in SSA, they take on a special importance”* without explaining why this is especially so.^8^

The article makes an important note of the need for a delineation of tasks between physicians and NPCs while working as a team. This is particularly important where physicians are present with NPCs at the same service delivery point. These scopes and the relative independence of practice of both NPCs and physicians shall depend on the circumstances and experience of each cadre type. An NPC practicing in a remote inaccessible area that has significant experience, more responsibility, and with the right training and experience may need less oversight than newly qualified physicians working independently for the first time. It is important to see the primary purpose of NPC roles as being about saving lives, and not as a threat to another profession’s turf.

A case was made in the article, that the presence of NPCs might have encouraged physicians’ to leave the public sector and may have fostered higher emigration. That the proposed training and curricula changes, to enable physicians deal with the NPC phenomenon will resolve a loss of doctors is rather speculative and difficult to accept. The solution proposed, ie, to train all physicians with managerial, mentoring, and supervisory skills, whether they will need them or not, will require in our view an expansion in curriculums already challenged by a double burden of disease including communicable diseases, non-communicable diseases (NCDs), lifestyle and behavioral conditions. The proposed curriculum changes are unlikely to be a responsible investment of resources, when most physicians may never use these skills and it is unlikely that physicians will lose their utility and influence over countries’ health systems because they lacked basic training of this kind. Indeed, health is a shared responsibility for all cadres and for the entire population.

Managerial and leadership roles in health systems have evolved and in many situations, health sector executives are public health and management trained and may not necessarily be health service professionals or doctors.

The proposal made by the article, for a reduction in current curriculum and coursework to accommodate the needs the authors describe, will be erroneous to pursue without a thorough and holistic review and understanding of the true needs of all cadres of health workers. With increasing urbanization, demographic changes and increasing incomes and changing lifestyles in SSA, that proposal if pursued, will conflict with a need to introduce much more important new physician skills needed to ensure enhanced roles and priority clinical skills that reflect the real changing health needs in their countries. SSA countries, currently with 80% of global NCD deaths (29 million out of 36 million deaths) should expand the scopes of all cadres including physicians to deal with the major challenges of the near future.^21^

In our debates to sustain health, we must all take care not to promote a culture of “Physician chauvinism” over the health sectors in SSA.

## Ethical issues

Not applicable.

## Competing interests

Authors declare that they have no competing interests.

## Authors’ contributions

DD wrote the first draft. IIKH and KO worked on the first draft, by substantiating and adding on to it. All authors revised the manuscript for important intellectual content. All authors read and approved the final version.

## Authors’ affiliations

^1^World Health Organization (WHO) Africa Region Office, Brazzaville, Congo. ^2^SUN Business Network, Global Alliance for Improved Nutrition, Abuja, Nigeria. ^3^Department of Health System Policies and Operations, World Health Organization Regional Office for Africa (AFRO) Brazzaville, Congo.
